# A novel CAF-related gene signature for prognostic and immunotherapy response prediction in PDAC based on single-cell RNA-seq and bulk RNA-seq data

**DOI:** 10.3389/fphar.2026.1873756

**Published:** 2026-10-09

**Authors:** Xuezhi Du, Zesheng Li, Yuwei Wang, Lei Han, Jinjin Sun, Weipeng Gong

**Affiliations:** 1 Department of Colorectal Surgery Ward I., Shandong Cancer Hospital and Institute, Shandong First Medical University and Shandong Academy of Medical Sciences, Jinan, Shandong, China; 2 Department of Hepatopancreatobiliary Surgery, The Second Hospital of Tianjin Medical University, Tianjin, China; 3 Key Laboratory of Post-Neuroinjury Neuro-Repair and Regeneration in Central Nervous System, Ministry of Education and Tianjin City, Tianjin Neurological Institute, Tianjin Medical University General Hospital, Tianjin, China; 4 Department of Critical Care Medicine, Shandong Provincial Hospital Affiliated to Shandong First Medical University, Jinan, Shandong, China

**Keywords:** cancer immunotherapy, cancer-associated fibroblasts, gene signature, immune infiltration, immunotherapy response, pancreatic ductal adenocarcinoma

## Abstract

Increasing evidence shows that cancer-associated fibroblasts (CAFs) contribute to tumor progression, create an immunosuppressive microenvironment, and promote resistance to immunotherapy. However, the CAF-related gene signature in pancreatic ductal adenocarcinoma (PDAC) remains largely unexplored. In our study, we constructed a nine-gene CAF-related signature and found that it strongly correlated with the prognosis of PDAC patients in different cohorts. Subsequently, we found that the CAF-related gene signature was associated with the genomic alteration landscape, immune infiltration landscape and responsiveness to immunotherapy. GJB2 had the highest coefficient in our CAF-related gene signature, thus we explored the role of GJB2 in the interactions between CAFs and PDAC cells. The results showed that conditioned medium (CM) derived from the GJB2 knockdown group could suppress the malignant phenotype of PANC1 and MIAPaCa2 cells. In summary, this CAF-related gene signature is useful for prognosis prediction and precision therapy of PDAC patients.

## Background

Pancreatic ductal adenocarcinoma (PDAC) is one of the most aggressive human cancers (Sung et al., 2021). Moreover, PDAC is expected to become the second leading cause of cancer-related mortality by 2040 (Halbrook et al., 2023). In addition, the majority of PDAC cases have no identifiable cause (Petersen, 2016), which makes PDAC prevention a challenge. Furthermore, owing to the difficulty of early diagnosis, unclear pathogenesis, and limited therapeutic options, the prognosis of PDAC patients is unsatisfactory (Halbrook et al., 2023). Over the last decade, omics technologies have developed rapidly providing us a new perspective of PDAC (Cao et al., 2021; Chen et al., 2021). Based on these omics data, a series of valuable multigene signatures were developed to predict the prognosis of PDAC (Yao et al., 2023; Yu et al., 2023; Zhang et al., 2023).

The tumor microenvironment (TME) includes various cellular components, and their interactions have been shown to contribute to the progression of different cancers (Zhang and Huang, 2011). Cancer-associated fibroblasts (CAFs) are highly abundant and are essential components of stromal cells in PDAC (Zaitsev et al., 2022). CAFs are derived from various cell types including fibroblasts or stellate cells (PSCs), mesenchymal stem cells (MSCs) or monocytes, pericytes, cancer cells, etc. CAFs can be classified into many different subgroups, which perform different functions (Brichkina et al., 2023; Rebelo et al., 2023). More precise classification of CAFs based on omics data is crucial for exploring their functions. Additionally, studies have shown that CAFs are closely linked to the prognosis of various types of cancers (Ge et al., 2022; Wang et al., 2023). Increasing evidence suggests that CAFs play a critical role in creating immunosuppressive microenvironments and contributing to therapeutic resistance (Han et al., 2020; Looi et al., 2019). Therefore, CAFs are very helpful for the prognosis prediction of cancer patients, and targeting CAFs is a new strategy for cancer treatment.

Although some bioinformatics analyses have been performed to investigate the subgroups of CAFs in PDAC (Zhang et al., 2022), their data were obtained from bulk RNA-seq rather than scRNA-seq. The scRNA-seq enables us to perform the analysis of RNA at the single-cell level, which can reveal intercellular heterogeneity and providing more precise biological information. In our study, several different CAFs subtypes were identified in PDAC, and a CAF-related gene signature was built according to a machine learning algorithm. Then, we systematically explored the relationships between the CAF-related gene signature and genomic alteration landscape, immune infiltration landscape and responsiveness to immunotherapy. Finally, we identified the key gene in the CAF-related signature and verified its impact on the interactions between CAFs and PDAC cells. In summary, our study developed a CAF-related gene signature that may offer new insights into the role of CAFs in PDAC development.

## Methods

### Data download and processing

The data of GSE212966 was obtained from GEO database. For single cell sequencing data, cells with more than 9,000 and less than 510 feature RNA, less than 1,100 count RNA and more than 20% mitochondrial RNA were removed. The PDAC bulk RNA-seq data of GSE28735 was obtained from GEO database. The differentially expressed genes (DEGs) of GSE28735 were analyzed by GEO2R. The corresponding data including clinical information, SNV and CNV data were acquired from TCGA database. The validation data of E-MTAB-6134 was obtained from ArrayExpress database. The validation data of PACA-AU was obtained from ICGC database.

### Identification of CAFs

The Seurat package was used to integrate, scale, cluster, and visualize scRNA data. Then the samples were merged and normalized. Next, we performed PCA analysis and top 25 PCs were selected according to the elbow plot. The selected PCs were used to conduct uniform manifold approximation and projection (UMAP) dimension reduction and cluster analysis with a resolution of 1.0 (Becht et al., 2018). CAF cells were annotated with ACTA2, PDGFRA, PDGFRB, and NOTCH3 (Yu et al., 2022; Chen et al., 2022). The annotated CAF cells were extracted and re-clustered. Marker genes in different CAF clusters were screened at the following criteria: threshold = 0.5, minpct = 0.35, and adj.*P*.value < 0.05. Specifically, iCAF scores were calculated using IL6, IL11, LIF, CXCL12, CXCL1, CXCL2, CXCL8, CCL2, CCL11, FAP, PDGFRA, PDPN, and HAS2. myCAF scores were calculated using ACTA2, TAGLN, MYH11, COL1A1, COL1A2, COL3A1, COL8A1, COL10A1, COL11A1, COL12A1, POSTN, MMP11, CTHRC1, and SDC1. Module scores were calculated using the AddModuleScore function in Seurat. The KEGG analysis of marker genes was performed with clusterProfiler package.

### Confirmation of CAF-related gene signature

The CAF marker genes were identified by the criteria of threshold = 0.5, minpct = 0.35, and adj.*P*.value < 0.05 according to the scRNA data. The DEGs were obtained from GSE28735. The genes included in both scRNA marker genes and DEGs were adopted for the subsequent analysis. We now describe the two-step selection procedure: univariate Cox screening (P < 0.05) followed by LASSO-Cox regression with 10-fold cross-validation, which identified nine-gene signature. LASSO cox regression analysis was performed to construct a CAF-related gene signature as following formula: Riskscore $\sum_{i=1}^{n}\left(\text{Expi}*\text{Coei}\right)$ . The i represents the gene included in the signature. Exp and Coe represent the expression level of the gene and the coefficient of this gene, respectively. The survival curves were plotted to compare OS between different groups by survival package. The ROC curve was performed to assess the predictive ability of Riskscore using timeROC package.

### Validation strategy

We clarify that 10-fold cross-validation was used during LASSO gene selection to reduce overfitting. External validation was conducted in independent GEO cohorts. Importantly, TCGA-derived Cox coefficients were applied without re-training in all validation cohorts. Model performance was assessed using time-dependent ROC (1-, 3-, and 5-year AUC) and Kaplan-Meier analysis.

### Normalization procedures

We now specify that both TCGA and GEO expression data were processed using log_2_ transformation log_2_(FPKM +1) for TCGA; Normalized expression values for GEO cohorts which the data is array to ensure cross-cohort comparability. Within each cohort, risk scores were z-score standardized before high/low risk classification (z-score > 0 = high risk).

### Clinical variables

We have added multivariate Cox regression adjusting for age, gender, stage, N stage, T stage and radiation therapy to demonstrate that the risk score remains an independent prognostic factor.

### Multi-omics alteration analysis

Tumor Mutation Burden (TMB) were calculated by maftools package (Mayakonda et al., 2018).The recurrent copy number alterations (CNA) including amplified and deleted regions were detected by GISTIC2.0 tools through GenePattern (https://www.genepattern.o rg/). The definition of fraction of genome alteration (FGA) was described in previous study (Lu et al., 2021).

### Pathway analysis of model genes and immune landscape analysis

The pathway analysis was performed by GSVA analysis. The status of immune cell infiltration were analysed by four tools, including “CIBERSORT” (Newman et al., 2015), “quanTIs eq” (Finotello et al., 2019), “MCP-counter” (Becht et al., 2016) and “Scaden” (Menden et al., 2020).

### Responsiveness to ICB therapy

The data of patients receiving anti-PD-L1 therapy were downloaded from the IMvigor210 cohort (Mariathasan et al., 2018). The data of patients receiving the anti-PD1 therapy were downloaded from GSE78220 datasets (Hugo et al., 2016). These two cohorts were used to predict responsiveness of ICB.

### Cell culture and siRNAs transfection

The primary CAF cells and normal tissue associated fibroblasts (NAF) were obtained from surgical specimens of pancreatic cancer patients using the previously established method (Meng et al., 2023). The patient signed an informed consent before the surgery. This study was approved by the Ethics Committee of the Second Hospital of Tianjin Medical University. The primary CAF cells were charactered by morphology and expression of αSMA and vimentin. The 10% fetal bovine serum (FBS) DMEM medium was used to culture CAFs, NAFs, PANC-1 and MIAPaCa-2 cell lines. The cells were grown at the environment of 37 °C and humidified atmosphere with 5% CO_2_. According to instructions, siRNAs were transfected into CAF cells.

### Immunofluorescence assay

Cell slides were put into 24-well plate, and 2 × 10^5^ CAF cells were seeded into each well. After 24 h, the cells were fixed for 15 min and incubate with 0.5% triton-100 for 20 min. Then the antibody of α-SMA (abcam ab5694) and vimentin (proteintech 60330-1-Ig) were diluted according to instructions and added into cell slides. After the primary antibody incubated overnight, fluorescent secondary antibody was incubated with samples in 37 °C for 90 min. Fluoroshield Mounting Medium with DAPI (ab104139) was used to cover the slides.

### Conditioned medium preparation

About 2 × 10^6^ CAF cells with or without knocking down GJB2 were put into 10 cm plates and cultured for 48 h. The medium was centrifuged and collected supernatant which can act as conditioned medium. PANC-1 and MIAPaCa-2 cell lines were treated with different conditioned medium (CM) to perform cytological experiments.

### RT-qPCR

The TRIzol (Takara, Japan) reagent was used to extract total RNA. The cDNA was generated by 2ug RNA using Reverse Transcription system. The expression level of mRNA was detected by qPCR master mix. The expression level of GAPDH used as control was measured in the same condition.

### CCK-8 and EDU assay

For CCK-8 experiment, 2 × 10^3^ PANC1 or MIAPaCa-2 cells resuspended with different CM were put into per well of 96-well plates. After arriving at the corresponding time point, the CM were discarded, and 10 μL CCK-8 reagent (APExBIO United States) mixed with 90 μL serum-free medium were added into per well. Next the plates were put into cell incubator for 1 h. Then, the OD values were detected at 450 nm. This process lasted for six consecutive days. For EDU assay, 2 × 10^5^ PANC1 or MIAPaCa-2 cells were seed into 96-well plates. After cultured with different CM, the assay was performed according to the EDU kit instructions.

### Transwell and wound healing assay

Before the assays, PANC1 or MIAPaCa-2 cells were pretreated with Mitomycin C to inhibit proliferation (Goulet et al., 2019). Then the upper chamber was put into a 24-well plate to perform Transwell assay. And 2 ×1 0^5^ PANC1 or MIAPaCa-2 cells resuspended with 200 μL CM without FBS were seeded into the upper chamber. 500 μL medium with 10% FBS was put into lower chamber. Forty-eight hours later, the cells were treated with 4% paraformaldehyde and 1% crystal violet solution. The upper chambers were cleaned gently to wipe off the remaining cells. The microscope (BX53, Olympus, Japan) was applied to collect the images. For wound healing assay, 2.5 × 10^6^ PANC1 or MIAPaCa-2 cells resuspended with different CM were seeded into per well of 6-well plate. After 48 h, the cells were scratched, and the medium was replaced with fresh CM without FBS The microscope (IX81, Olympus, Japan) was applied to collect the images at 0h, 24 and 36 h. Three replications were performed for each assay.

### Statistical analysis

The R software (v4.2.2) was used to perform statistical analyses. Pearson or Spearman correlations were used for the correlation matrices. In order to compare the two groups, a Wilcoxon test was conducted.

## Statements

The data of this study was obtained from the public databases and all methods followed the relevant guidelines and regulations.

## Results

### Confirmation of CAFs

The scRNA data of GSE212966 were analyzed by the Seurat R package. After preprocessing, the influence of mitochondrial RNAs was removed (Supplementary Figures S1A–C). The top 25 principal components (PCs) were selected by linear dimensionality reduction (Supplementary Figure S1D). A total of thirty-one clusters were obtained from UMAP dimensionality reduction (Figure 1A). The CAF clusters were annotated by ACTA2, PDGFRA, PDGFRB, and NOTCH3 (Yu et al., 2022; Chen et al., 2022). The distribution of four marker genes and expression levels around thirty-one clusters are shown in Figure 1B and Supplementary Figure S1E. Then, seven CAF clusters are obtained by further dimension reduction (Figure 1C). The distribution of four marker genes around the seven CAF clusters is shown in Figure 1D. The marker-expression DotPlot and module-score analyses demonstrated that clusters 4, 5 showed enrichment of the iCAF signature, including inflammatory cytokines/chemokines and canonical markers, and were therefore annotated as iCAFs. In contrast, clusters 0, 2, 6 showed enrichment of the myCAF signature, characterized by high expression of contractile and extracellular-matrix-related genes, including ACTA2, TAGLN, MYH11, COL1A1, COL1A2, POSTN, and CTHRC1 and were annotated as myCAFs (Supplementary Figures S1F–H).

**FIGURE 1:**
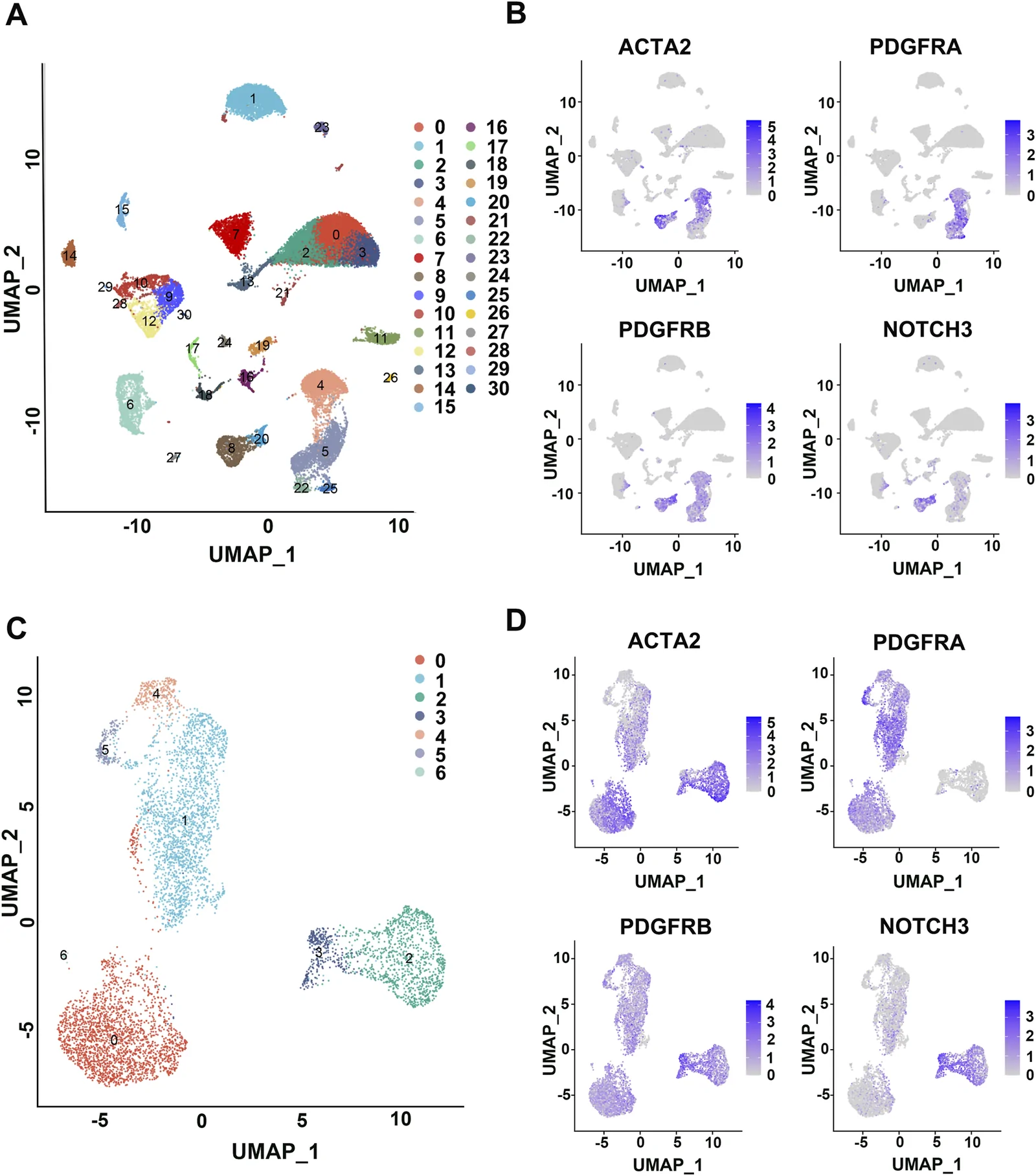
Confirmation of CAF-related clusters from PDAC single-cell RNA-sequencing data. **(A)** Visualization of clusters after uniform manifold approximation and projection (UMAP) reduced dimensionality in PDAC samples (GSE212966). **(B)** The distribution of four CAF marker genes of CAFs in the UMAP plot. **(C)** Visualization of 7 CAF-related clusters after further UMAP reduced-dimensionality. **(D)** The distribution of four CAF marker genes in all 7 clusters in the UMAP plot.

The UMAP plot of CAFs in the samples from GSE212966 is shown in Figure 2A. The UMAP plot of CAFs in normal and tumor sample distributions is shown in Figure 2B. Cluster 4 and Cluster 5 were mainly distributed in the normal group, while Cluster 0 was mostly observed in the tumor group. A total of 1,587 marker genes across 7 CAF clusters were identified. The top 6 marker genes for each CAF cluster are shown in Figure 2C. Figure 2D illustrated the proportions of the 7 CAF clusters in each sample. Samples T2 and N2 exhibited the highest number of CAF cells in malignant and nonmalignant tissues, respectively. In sample T2, Cluster 0 had the highest proportion, while in sample N2, Cluster 1 was most predominant. Based on CNV characteristics, the cells in 7 CAF clusters were categorized into malignant and nonmalignant cells (Figure 2E). Cluster 2 exhibited a higher proportion of malignant cells. KEGG analysis revealed that the 7 CAF clusters display functional heterogeneity, indicating differences in their physiopathological roles. Cluster 2 is primarily involved in carcinogenic pathways, including the MAPK signaling pathway, TNF signaling pathway, and cGMP-PKG signaling pathway (Figure 2F).

**FIGURE 2:**
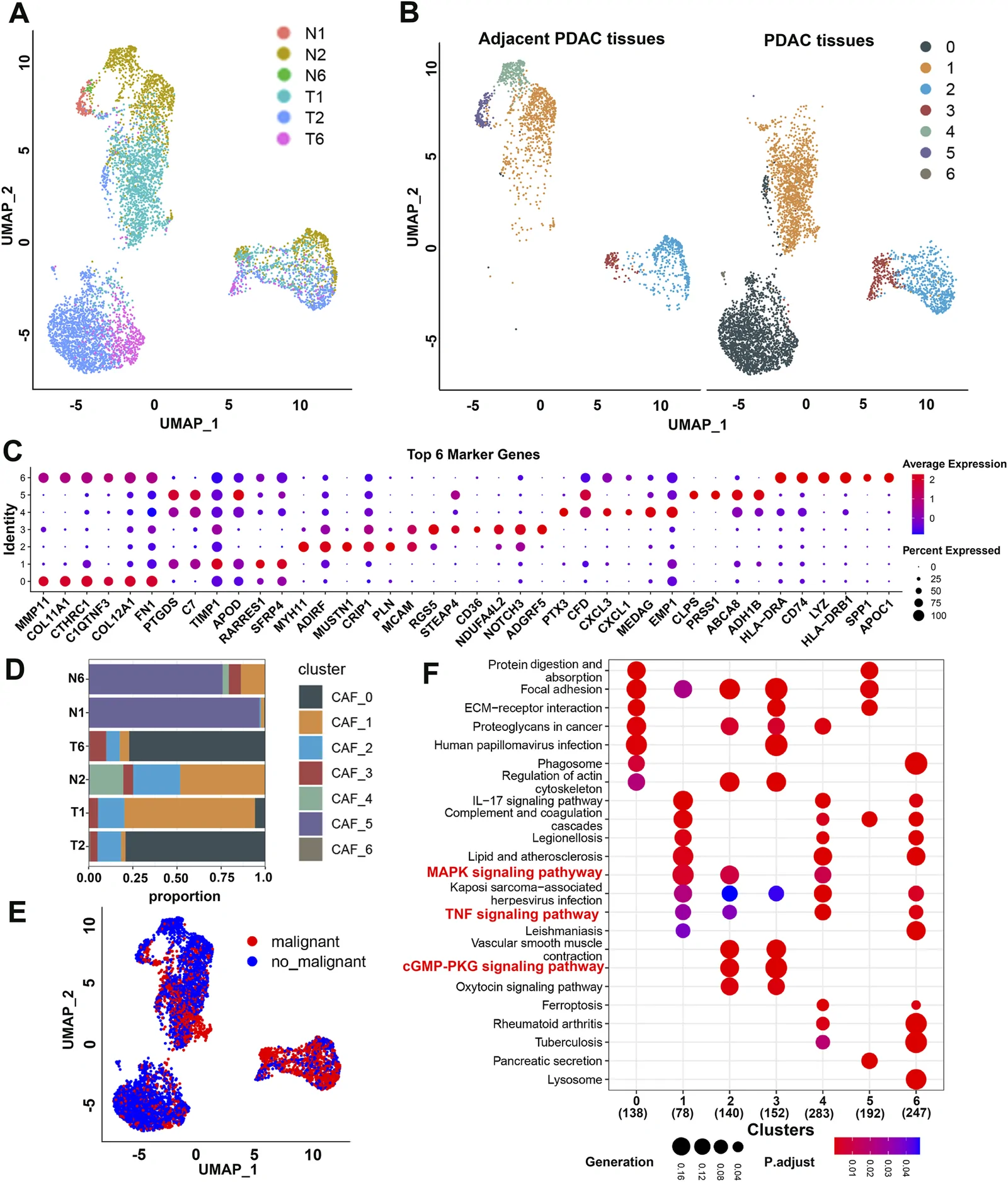
Analysis of the characteristics of CAF-related clusters in PDAC samples. **(A)** UMAP plot of CAF-related clusters in three pairs of samples containing PDAC and adjacent PDAC tissues from the GSE212966 dataset. **(B)** The distributions of CAF-related clusters in PDAC and adjacent PDAC tissues. **(C)** The expression of CAF-related cluster marker genes. **(D)** The proportion of the 7 CAF-related clusters in three pairs of samples containing PDAC and adjacent PDAC tissues. **(E)** UMAP plot of malignant and nonmalignant cells calculated by the copykat package in three pairs of samples containing PDAC and adjacent PDAC tissues. **(F)** KEGG enrichment analysis of marker genes from 7 CAF-related clusters.

### Prognosis analysis of CAF clusters

To evaluate the association between CAF clusters and prognosis, we calculated the ssGSEA scores for the top 5 marker genes of each CAF cluster. Using the optimal cutoff value, PDAC samples were then categorized into distinct groups (Yu et al., 2022). Samples with high CAF scores were associated with poorer prognoses in Clusters 0 (Figure 3A), 1 (Figure 3B), 4 (Figure 3E), and 6 (Figure 3G). In contrast, samples with low CAF scores had a worse prognosis only in Cluster 5 (Figure 3F). No significant correlation with prognosis was observed in Clusters 2 (Figure 3C) and 3 (Figure 3D).

**FIGURE 3:**
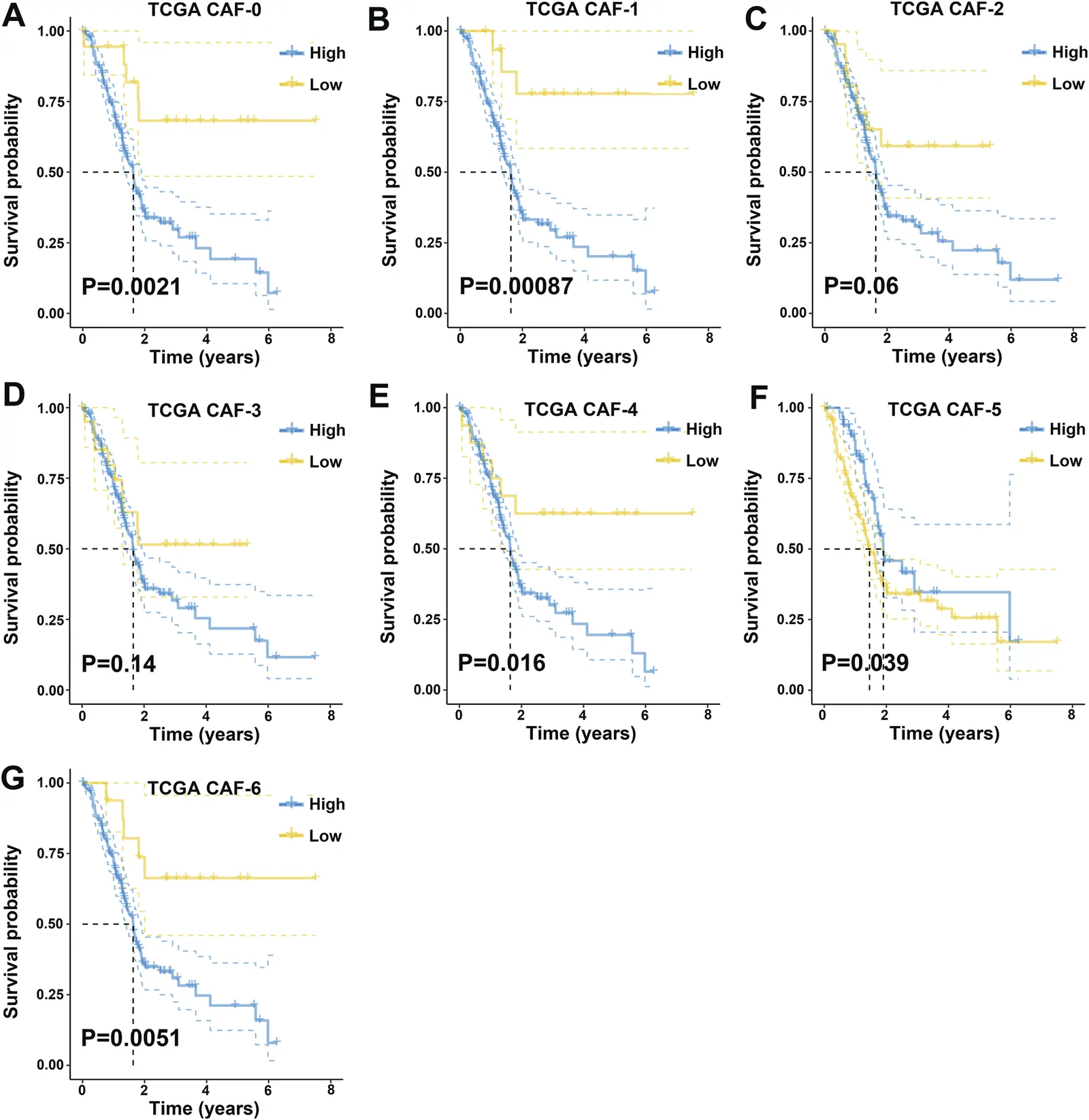
The relationship between 7 CAF-related clusters and the prognosis of PDAC patients from the TCGA dataset. The survival curves of different CAF score groups in the CAF 0 cluster **(A)**, CAF 1 cluster **(B)**, CAF 2 cluster **(C)**, CAF 3 cluster **(D)**, CAF 4 cluster **(E)**, CAF 5 cluster **(F)** and CAF 6 cluster **(G)**.

### Identification of CAF-related gene signature

First, we screened out differentially expressed genes (DEGs) between normal and tumor samples in GSE28735 (Figure 4A). A total of 440 DEGs (adj.*P*.value < 0.05, |log2FC|≥1) were obtained (Supplementary Table S2). In addition, 1,587 DEGs among the 7 CAF clusters were identified in the above analysis (Supplementary Table S1). A total of 116 CAF-related DEGs were identified by intersecting CAF cluster marker genes with DEGs (Figure 4B). Eleven CAF-related DEGs demonstrated prognostic significance (Figure 4C). After LASSO Cox regression analysis, nine genes remained at lambda = 0.0264 (Figures 4D,E). Finally, a nine gene CAF-related gene signature, including GJB2, SULF1, LMO3, AHNAK2, PLAT, FOSB, NT5E, CLPS and ADAMTS12, was built (Figure 4F). The formula of the CAF-related gene signature is shown below:

$$\text{Riskscore}=\left(0.516\times \text{GJB}2\right)+\left(0.121\times \text{SULF}1\right)+\left(0.091\times \text{LMO}3\right)+\left(0.077\times \text{AHNAK}2\right)+\left(0.051\times \text{PLAT}\right)+\left(-0.005\times \text{FOSB}\right)+\left(-0.022\times \text{NT}5E\right)+\left(-0.253\times \text{CLPS}\right)+\left(-0.286\times \text{ADAMTS}12\right)$$

**FIGURE 4:**
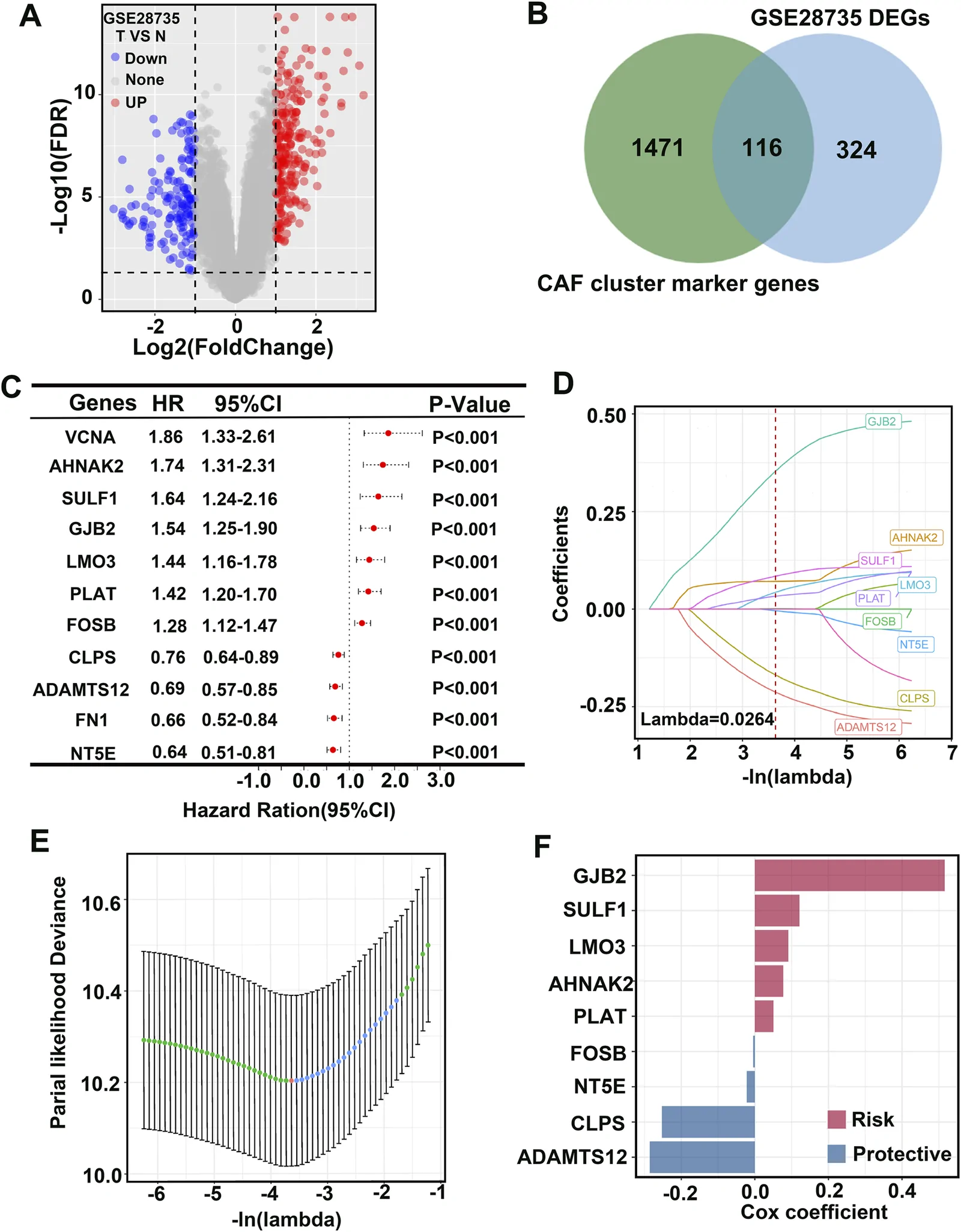
Confirmation of CAF-related gene signature. **(A)** Volcano plot of DEGs “obtained from GSE28735”. **(B)** Venn plot of CAF-related DEGs. **(C)** Forest plot of the results from the univariate Cox regression analysis. **(D)** Plot of lambda trajectories for the independent variables. **(E)** Distributions of coefficients for the logarithmic (lambda) series for parameter selection. **(F)** Plot of coefficients of the CAF-related gene signature.

Samples were then classified into different risk groups based on their Riskscore after z-score normalization in the training set (TCGA) and validation sets (E-MTAB-6134 and PACA-AU). Patients in the low-risk group had a better prognosis in the TCGA training set (Figure 5A). This trend was similarly observed in the E-MTAB-6134 (Figure 5C) and PACA-AU validation sets (Figure 5E). In the TCGA cohort, the AUC values were 0.70 for 1 year, 0.79 for 3 years, and 0.89 for 5 years (Figure 5B). In the E-MTAB-6134 cohort, the AUC values were 0.77 for 1 year, 0.65 for 3 years, and 0.65 for 5 years (Figure 5D). In the PACA-AU cohort, the AUC values were 0.70 for 1 year, 0.67 for 3 years, and 0.62 for 5 years (Figure 5F). These results indicate that the CAF-related gene signature demonstrates robust and reliable performance in predicting the prognosis of PDAC patients.

**FIGURE 5:**
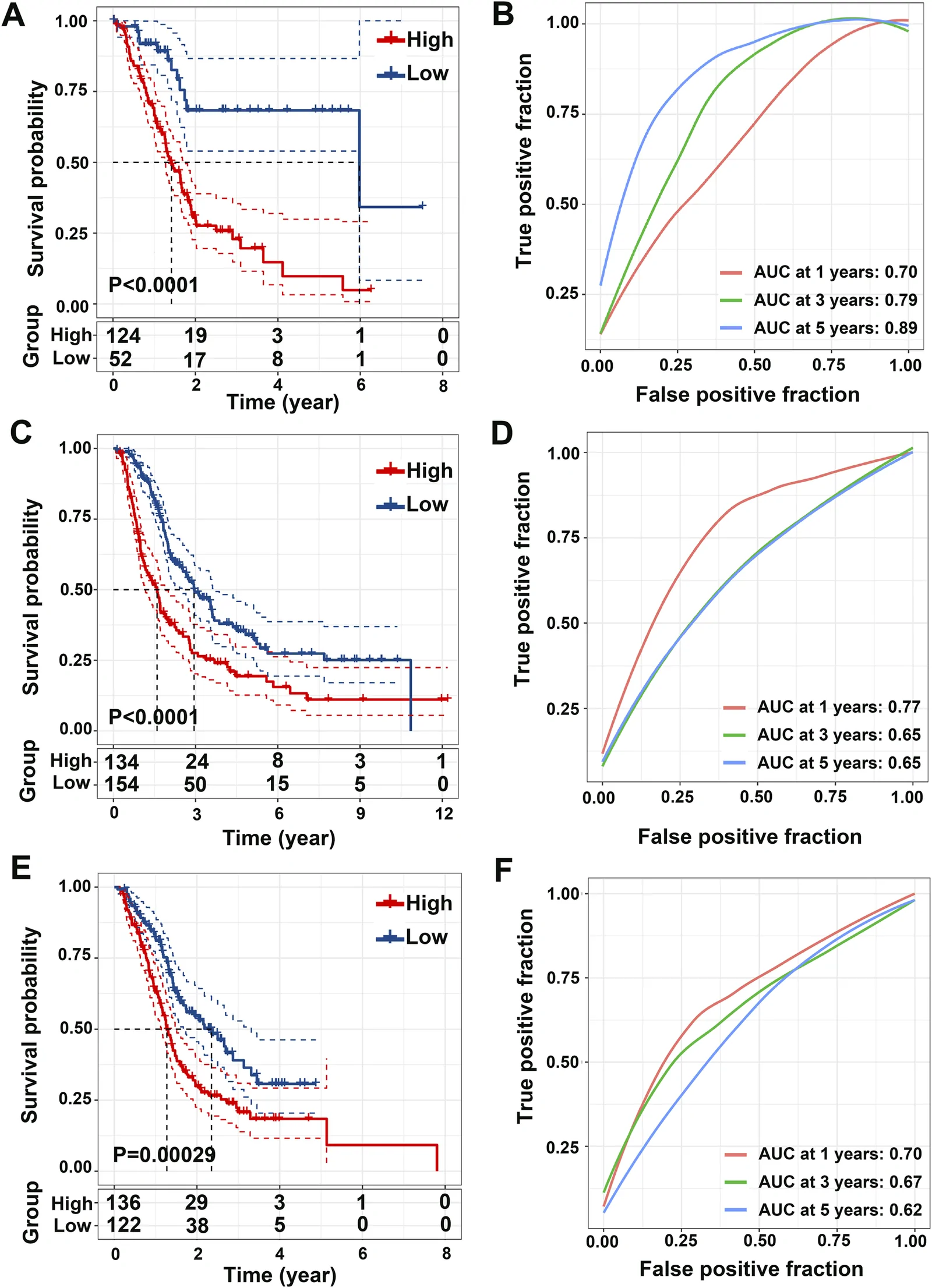
Prognostic performance of the CAF-related gene signature. The survival curve **(A)** and ROC curve **(B)** of the CAF-related gene signature in the TCGA cohort. The survival curve **(C)** and ROC curve **(D)** of the CAF-related gene signature in the E-MTAB-6134 validation set. The survival curve **(E)** and ROC curve **(F)** of the CAF-related gene signature in the PACA-AU validation set.

### Correlations between CAF-related gene signature and multiomics alterations

Significant differences in multiomics were observed between the different groups. Mutations in KRAS, TP53, SMAD4, and CDKN2A contribute to the development of PDAC (Hu et al., 2021). Our results showed that these four genes had the highest mutation frequency in the TCGA cohort (Figure 6, upper panel). In addition, the mutation frequencies of KRAS, TP53, SMAD4, and CDKN2A were higher in the high-risk group (Supplementary Figure S2A,B). In addition to the higher amplification frequency of SMAD4, CDKN2A and TP53 observed in the low-risk group, the amplification frequency of KRAS was not significantly different (Figure 6, lower panel). Increasing evidence has shown that chromosomal instability can lead to poor prognosis in various cancers (Paolini et al., 2022; Comaills and Castellano-Pozo, 2023). In our study, we found that the frequency of chromosomal arm amplification or deletion was higher in the high-risk group, indicating high chromosomal instability (Figure 6, middle panel; Supplementary Figure S2C,D).

**FIGURE 6:**
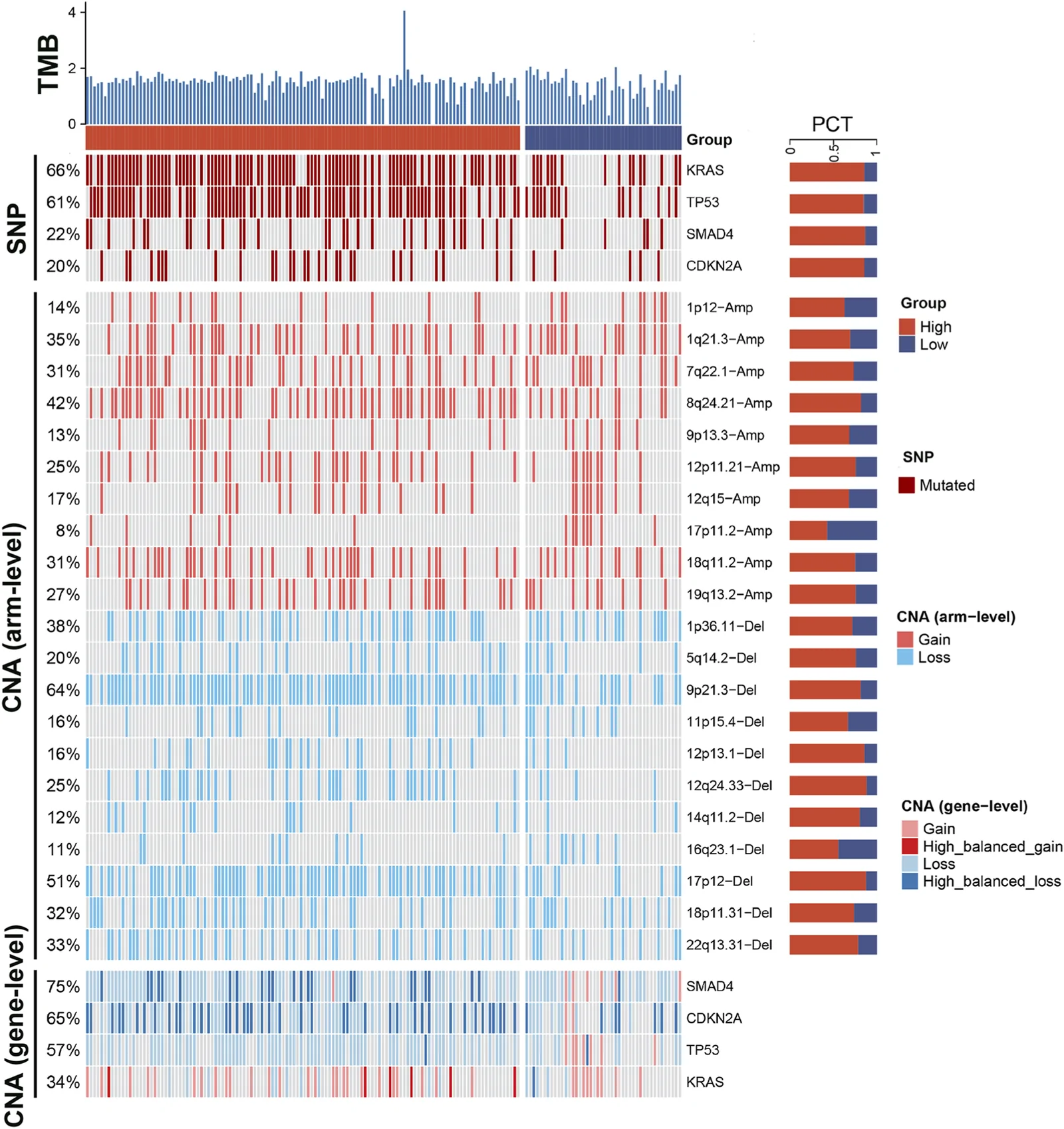
The landscape of genomic alterations of high-risk group and low-risk group in TCGA cohort. Upper panel: Heatmap of the mutation frequency of KRAS, TP53, SMAD4 and CDKN2A in the high and low risk groups of the TCGA PDAC dataset. Middle panel: Heatmap of copy number alterations (CNAs) at the chromosome arm level in the high and low risk groups of TCGA PDAC dataset. Lower panel: Heatmap of copy number alterations (CNAs) of KRAS, TP53, SMAD4 and CDKN2A in the high and low risk groups of TCGA PDAC dataset.

### Relationship between CAF-related gene signature and pathway

We explored the pathways associated with each risk signature gene. Twenty-three pathways were linked to these genes. The risk genes (GJB2, SULF1, LMO3, AHNAK2, and PLAT) were primarily associated with cancer-related pathways, including those involved in small cell lung cancer, pathways in cancer, and renal cell carcinoma. In contrast, most protective genes (NT5E, CLPS, and ADAMTS12) were related to other pathways, such as the citrate cycle/TCA cycle and oxidative phosphorylation. Significant differences were observed in the pathways associated with risk versus protective genes (Figure 7; Supplementary Figure S3).

**FIGURE 7:**
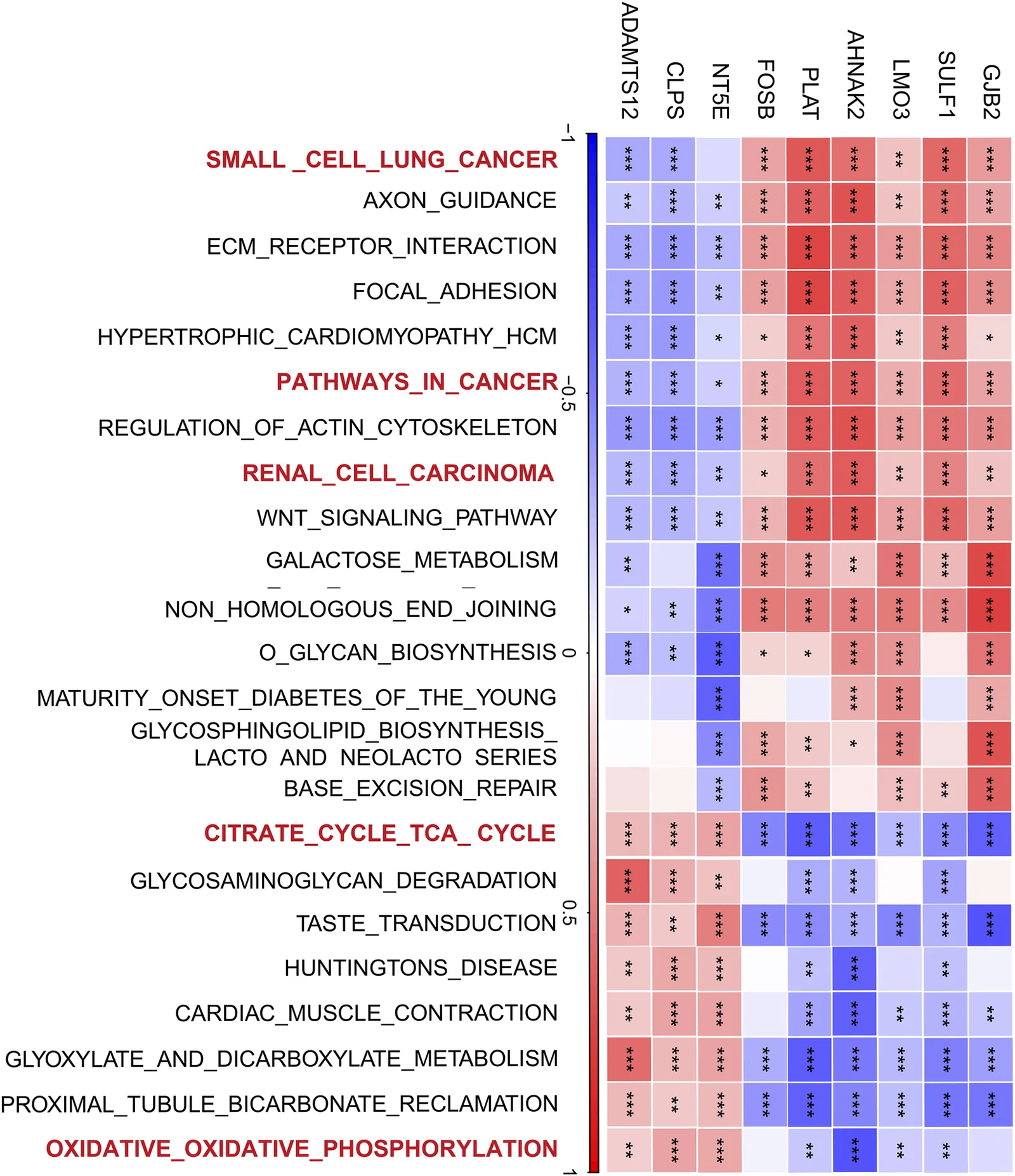
The heatmap of correlation between pathways and the genes in the CAF-related gene signature in the TCGA PDAC cohort.

### Relationship between CAF-related gene signature and immunity

It has been reported that CAFs can modulate immune cell infiltration in pancreatic cancer (Stouten et al., 2023). Therefore the methods of “CIBERSORT” (Newman et al., 2015), “quanTIseq” (Finotello et al., 2019), “MCP-counter” (Becht et al., 2016) and “Scaden” (Menden et al., 2020) were used to explore the effect of the CAF-related gene signature we constructed on immune cell infiltration in PDAC. We observed that CD4 T cells, CD8 T cells, and lymphocytes were reduced in the high-risk group according to all four algorithms (Figure 8; Supplementary Figure S4A–D). The decrease in T cell cytotoxicity and infiltration may contribute to the poor prognosis in high-risk group patients.

**FIGURE 8:**
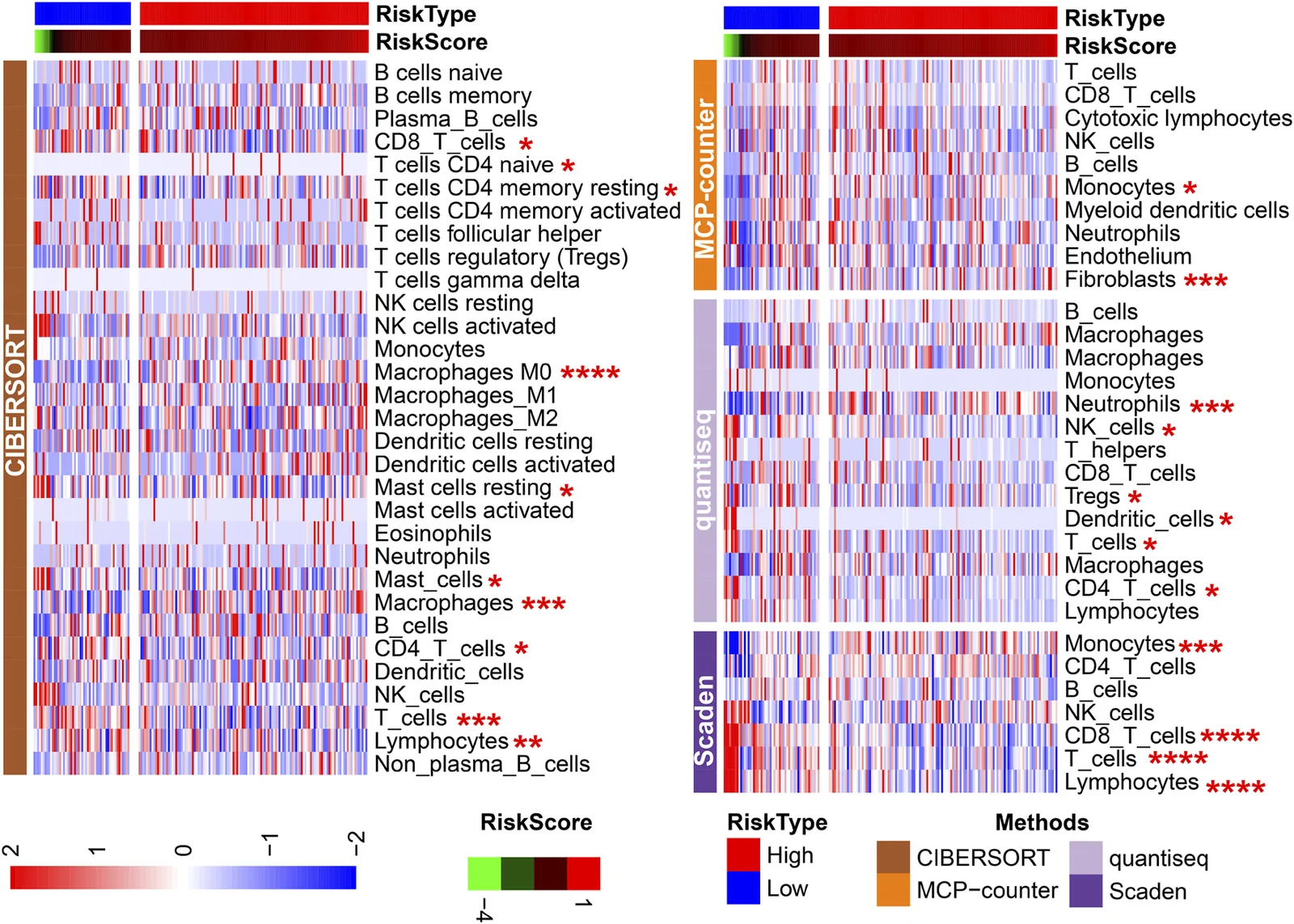
The difference in the immune landscape between different groups in the TCGA cohort. The heatmap of immune infiltrating cell status estimated by “CIBERSORT”, “quanTIseq”, “MCP-counter” and “Scaden” between high- and low-risk groups in the TCGA cohort.

### The relationship between the CAF-related gene signature and ICB therapy responsiveness

The predictive value of the CAF-related gene signature for ICB therapy was assessed in the IMvigor210 and GSE78220 cohorts. We observed that high-risk group patients had a worse prognosis in the IMvigor210 cohort (Figure 9A). We then explored whether the results differed by stage. The survival curves for patients in stages I + II and III + IV are shown in Figures 9B,C, respectively. In conclusion, the Riskscore provides a more accurate prognosis evaluation in advanced patients. Consistently, patients in the high-risk group had a worse prognosis. Additionally, we observed varied responses to ICB therapy among different groups in the IMvigor210 cohort. PD/SD patients had a higher Riskscore compared to CR/PR patients (Figure 9D). A higher percentage of PD/SD was observed in the high-risk group (Figure 9E). In the GSE78220 cohort, high-risk group patients also had a worse prognosis (Figure 9F), and PD/SD patients had a higher Riskscore compared to CR/PR patients (Figure 9G). A higher percentage of PD/SD was observed in the high-risk group (Figure 9H). These results illustrated that high-risk group patients exhibit poorer responsiveness to immunotherapy across different cancers, highlighting the broad applicability of our risk signature.

**FIGURE 9:**
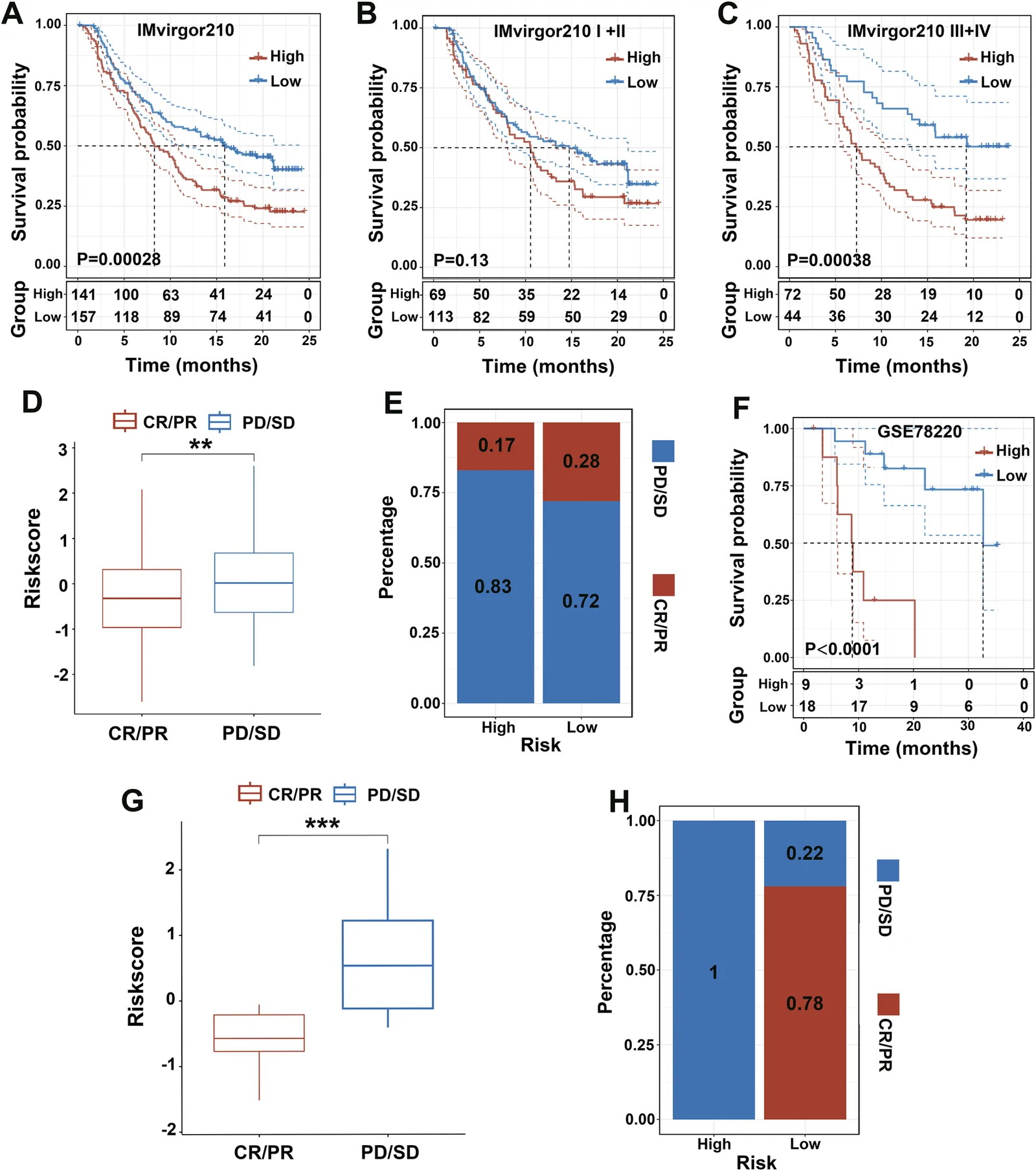
Prediction of responsiveness to immune checkpoint blocks based on Riskscore. The difference in survival rate between different groups in all patients in the IMvigor210 cohort **(A)**, early stage patients in the IMvigor210 cohort **(B)** and advanced stage patients in the IMvigor210 database **(C)**. **(D)** Box plot of Riskscore difference between the CR/PR and PD/SD groups in the IMvigor210 cohort. **(E)** The difference in immunotherapy responses between different groups in the IMvigor210 cohort. **(F)** The difference in survival rate between the high- and low-risk groups in the GSE78220 database. **(G)** Box plot of Riskscore difference between the CR/PR and PD/SD groups in the GSE78220 database. **(H)** The difference in immunotherapy responses between different groups in the GSE78220 database. CR: Complete Response PR: Partial Response PD: Progressive Disease SD: Stable Disease.

### The biological functions of GJB2 for interactions between CAFs and PDAC cells

Previous studies have found that CAFs can interact with PDAC cells, and conditioned medium (CM) derived from CAFs subjected to different treatments can affect the malignant phenotype of tumor cells (Meng et al., 2023; Zheng et al., 2022; Hu et al., 2022). GJB2 had the highest coefficient among the model genes and was explored in PDAC. Therefore, we further investigated the biological function of GJB2 in the interactions between CAFs and PDAC cells. NAFs and CAFs were obtained from surgical specimens of PDAC patients. Immunofluorescence results showed that both NAFs and CAFs express vimentin, but αSMA expression was significantly stronger in CAFs than in NAFs (Supplementary Figure S5). These findings confirm the successful acquisition of CAFs and NAFs from surgical specimens. (Ogier et al., 2018). In subsequent analyses, we found that GJB2 expression levels were higher in CAFs than in NAFs (Figure 10A). We then knocked down GJB2 in CAFs (Figure 10B) and obtained conditioned medium (CM) from different groups to treat PDAC cells. The results showed that cell proliferation was inhibited after treatment with CM from the GJB2 knockdown groups (Figures 10C–E). Wound healing assays demonstrated that the migration ability of PANC-1 and MIAPaCa-2 cell lines was suppressed when cultured with CM from GJB2 knockdown groups (Figures 10F,G). A similar result was observed in the transwell assay (Figure 10H). In conclusion, these results indicate that CM derived from GJB2 knockdown CAFs can inhibit the malignant phenotypes of pancreatic cancer cells.

**FIGURE 10:**
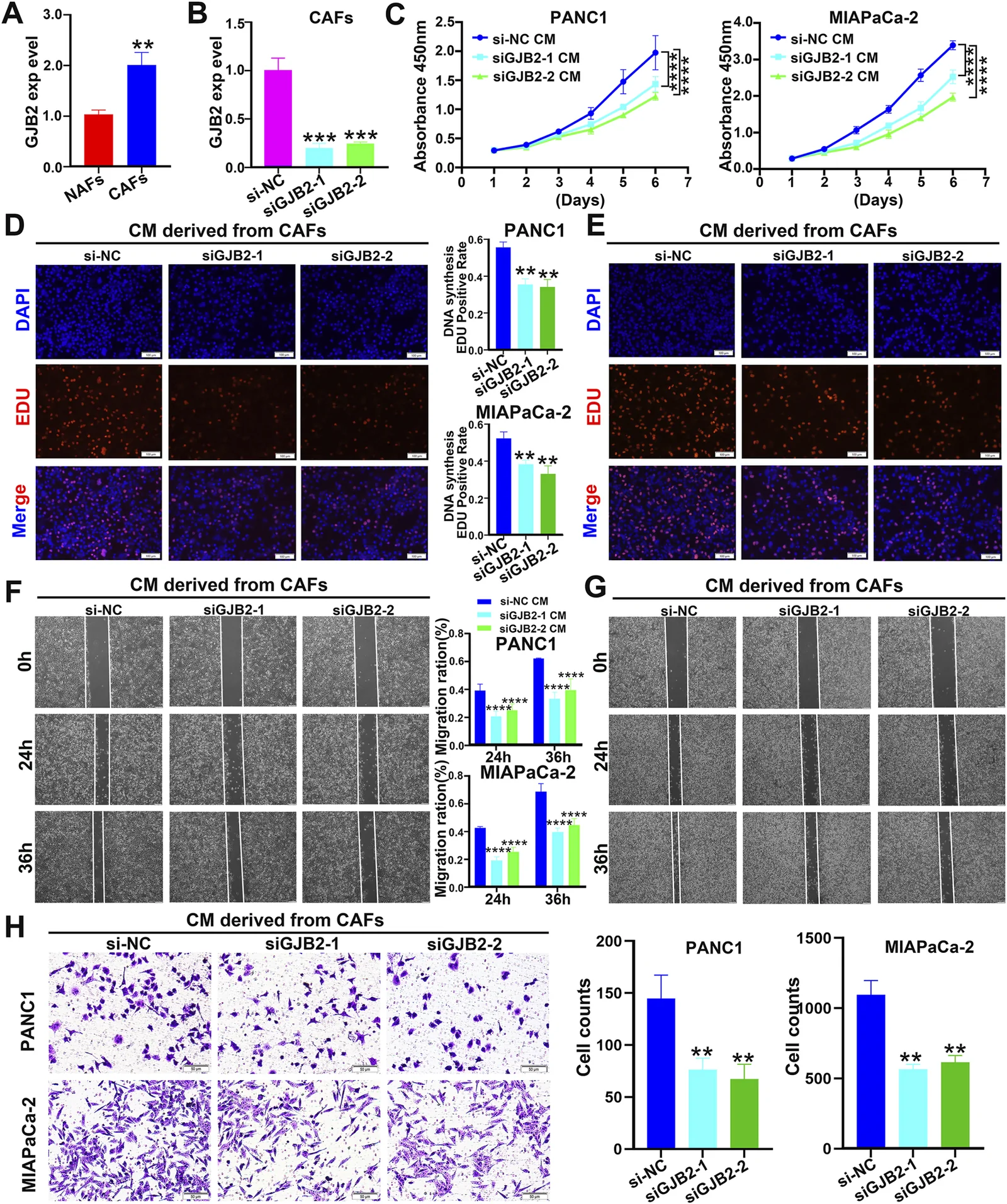
The biological functions of GJB2 in PDAC. **(A)** The expression level of GJB2 in NAFs and CAFs. **(B)** The knockdown efficiency of GJB2 in CAFs. **(C)** The influence of CM from GJB2 knockdown groups on the proliferation of PDAC cell lines detected by CCK-8. The influence of CM from GJB2 knockdown CAF groups on the proliferation of PANC1 cells **(D)** and MIAPaCa-2 cells **(E)** detected by EDU assay. The results of the wound healing assay of PANC1 cells **(F)** and MIAPaCa-2 cells **(G)** after treated with CM from GJB2 knockdown CAF groups. **(H)** The results of Transwell after PANC1 cells and MIAPaCa-2 cells treated with CM from GJB2 knockdown CAF groups.

## Discussion

In addition to tumor cells, the tumor microenvironment (TME) plays a crucial role in cancer development. CAFs are key components of the TME and create a supportive environment for tumor cells. CAFs can remodel the TME to promote metastasis, therapeutic resistance, dormancy, and reactivation (Chhabra and Weeraratna, 2023). Additionally, CAFs are highly abundant in the TME of pancreatic ductal adenocarcinoma (PDAC) (Zaitsev et al., 2022). With growing interest in CAFs, numerous CAF-related signatures have been developed due to advancements in omics technologies. Additionally, advances in scRNA-seq technology have enhanced our understanding of CAFs. Therefore, constructing a more precise CAF-related gene signature using scRNA-seq data is both feasible and urgent.

In this study, we identified 7 CAF clusters using scRNA-seq data. Our results revealed that the marker genes of these CAF clusters are associated with several cancer-related pathways, including focal adhesion, MAPK, and TNF signaling pathways. Subsequently, we developed a robust and reliable 9-gene CAF-related signature using a machine learning algorithm. Among these nine genes, GJB2, SULF1, LMO3, AHNAK2, and PLAT acted as risk factors, while FOSB, NT5E, CLPS, and ADAMTS12 served as protective factors. Next, we identified 23 pathways significantly correlated with the nine model genes. The risk genes were mainly linked to cancer pathways such as small cell lung cancer, renal cell carcinoma, the WNT signaling pathway, ECM-receptor interaction, focal adhesion, and pathways in cancer. In contrast, most of the protective genes were associated with non-tumor pathways, including glycosaminoglycan degradation, glyoxylate and dicarboxylate metabolism, the citrate cycle TCA cycle, and oxidative phosphorylation. These protective genes were closely linked to several metabolic pathways, suggesting a potential direction for further research. Our study showed that these nine model genes are differentially expressed in PDAC and are associated with prognosis, and their specific roles require further investigation.

The survival curves indicated that the high-risk group had a worse prognosis in both the training set and the validation sets. Thus, the Riskscore can serve as a prognostic marker for overall survival (OS) in PDAC patients. The ROC curves demonstrated that the Riskscore effectively predicted 1-, 3-, and 5-year OS in PDAC patients. The robust and reliable performance of the CAF-related risk model across different cohorts demonstrates its significant potential for clinical application.

It has been reported that cross-talk between CAFs and the tumor immune microenvironment (TIME) can promote cancer progression (Barrett and Puré, 2020). In this study, immune cell infiltration levels were assessed using four different methods. The results revealed variations in the abundance of NK cells, macrophages, fibroblasts, and T cells across the groups. All these cell types are associated with tumor immunity in immunotherapy (Chen et al., 2023; Stojanovic and Cerwenka, 2011; Want et al., 2023; Zhuang et al., 2023). Thus, we hypothesized that patients in the two groups might respond differently to immunotherapy. To test this hypothesis, we developed the CAF-related gene signature using the IMvigor210 and GSE78220 databases, which focus on ICB therapy. The Riskscore exhibited a negative correlation with OS in these immunotherapy datasets. Additionally, the results indicated that patients in the low-risk group may benefit more from ICB therapy. Due to the lack of an ICB therapy database for pancreatic cancer, we used the IMvigor210 database from bladder cancer and the GSE78220 database from melanoma to conduct a preliminary validation of the gene signature efficiency. This has certain limitations. The role of this model in evaluating the ICB effect of pancreatic cancer should be further explored in the future.

Previous studies have shown that CAFs could interact with PDAC cells, and conditioned medium (CM) from CAFs treated with different therapies can affect the malignant phenotype of tumor cells (Meng et al., 2023; Stouten et al., 2023; Zheng et al., 2022). GJB2 had the highest coefficient around the model genes, and it was poorly studied in PDAC. Therefore, we further investigated the biological function of GJB2 in the interactions of CAFs and PDAC cells. GJB2 is also known as Connexin 26, which contributes to form gap junction (Li et al., 2022). It has been reported that GJB2 can promote tumorigenesis and the progression of various cancers, including breast cancer (Liu et al., 2019; Thiagarajan et al., 2018) and NSCLC (Zeng et al., 2019). In another study, Li et al. found that GJB2 can influence the radiosensitivity of hepatocellular carcinoma cells (Li et al., 2022). In addition, GJB2 is also reported to serve as a biomarker in PDAC (Sun et al., 2018) and cervical cancer (Meng et al., 2022). In our study, CM from GJB2 knockdown CAFs inhibited the proliferation and migration of PANC-1 and MIAPaCa-2 cells. Our study showed that GJB2 can influence the interaction between CAFs and PDAC cells, and is valuable for further study.

Compared with previous studies, our research jointly analyzed single-cell RNA-seq and bulk RNA-seq data, which made the model more convincing. Furthermore, we explored the mechanism by which CAF exerts its effects at the cellular level, which provided a direction for subsequent research. However, some shortcomings exist in this study. First, CAFs may not be fully accurately annotated due to the current state of scRNA-seq technology. This can lead to a mismatch between our CAF- related gene signature and the actual situation. Second, our data were obtained from public databases and are retrospective. A multicenter and prospective cohort study is needed to estimate the prognostic value of the CAF-related signature. Thirdly, this study only investigated the role of GJB2 at the cellular level, and the impact of GJB2 on the response of pancreatic cancer to ICB remains unclear. In the future, more comprehensive mechanism exploration should be conducted. For instance, *in vivo* tests could be conducted on pancreatic cancer mouse models with normal immune functions. Finally, while the study demonstrates predictive performance, it does not yet establish clinical benefit or decision utility in prospective clinical workflows.

## Conclusion

In conclusion, we constructed a CAF-related gene signature which had a great potential for clinical application.

## Data Availability

The data presented in the study are deposited in publicly available databases. The scRNA-seq data GSE212966 and bulk RNA-seq data GSE28735 are available in the GEO database. The corresponding clinical information, SNV, and CNV data are available in the TCGA/GDC database (https://portal.gdc.cancer.gov/projects/TCGA-PAAD). The validation dataset E-MTAB-6134 is available in the ArrayExpress/BioStudies database (https://www.ebi.ac.uk/biostudies/arrayexpress/studies/E-MTAB-6134). The validation dataset PACA-AU is available in the ICGC database (https://dcc.icgc.org/projects/PACA-AU).

## Supplementary material

SUPPLEMENTARY FIGURE 1 Identification of CAF‐related clusters from PDAC scRNA sequencing data. **(A)** Plot of correlations between mRNA quantity and readings. **(B)** Plot of correlations between the proportion of mitochondrial genes and mRNA readings. **(C)** Plot of correlations between the proportion of mitochondrial genes and mRNA quantity. **(D)** Elbow plot of linear dimensionality reduction analysis of principal components. **(E)** The plot shows the distributions of CAF marker genes in 31 clusters. **(F)** The dotplot of CAF subtype markers across clusters. **(G)** The violin plots of iCAF and myCAF score. **(H)** The UMAP visualizations of iCAF and myCAF module score.

SUPPLEMENTARY FIGURE 2 The landscape of genomic alterations in the high-risk group and low-risk group. The plots of somatic mutations in the high-risk group **(A)** and low-risk group **(B)** of PDAC patients in the TCGA cohort. The plots of amplification frequency **(C)** and deletion **(D)** between different groups in the TCGA cohort.

SUPPLEMENTARY FIGURE 3 The heatmap of enrichment scores for key pathways in which the model genes were involved.

SUPPLEMENTARY FIGURE 4 The difference in the immune landscape between different groups in the TCGA cohort. Box plot of immune infiltrating cells of different groups in the TCGA cohort identified by CIBERSORT **(A)**, quanTIseq **(B)**, MCP-counter **(C)** and Scaden **(D)**.

SUPPLEMENTARY FIGURE 5 The expression of αSMA and vimentin in NAFs and CAFs.
